# Effect of Women’s autonomy on maternal health service utilization in Nepal: a cross sectional study

**DOI:** 10.1186/s12905-016-0305-7

**Published:** 2016-05-13

**Authors:** Ramesh Adhikari

**Affiliations:** Geography and Population Department, Mahendra Ratna Campus, Tribhuvan University, P.O.Box 1048, Kathmandu, Nepal

**Keywords:** Women’s autonomy, Maternal health, Cross sectional, Nepal

## Abstract

**Background:**

Women’s role has been a priority area not only for sustainable development, but also in reproductive health since ICPD 1994. However, very little empirical evidence is available about women’s role on maternal health service utilization in Nepal. This paper explores dimensions of women’s autonomy and their relationship to utilization of maternal health services.

**Methods:**

The analysis uses data from the Nepal Demographic and Health Survey, 2011. The analysis is confined to women who had given birth in the 5 years preceding the survey (n = 4,148). Women’s autonomy related variables are taken from the standard DHS questionnaire and measured based on decision in household about obtaining health care, large household purchases and visit to family or relative. The net effect of women’s autonomy on utilization of maternal health services after controlling for the effect of other predictors has been measured through multivariate logistic regression analysis.

**Results:**

The findings indicate only about a half of the women who had given birth in the past 5 years preceding the survey had 4 or more ANC check up for their last birth. Similarly, 40 % of the women had delivered their last child in the health facilities. Furthermore, slightly higher than two-fifth women (43 %) had postnatal check up for their last child. Only slightly higher than a fourth woman (27 %) had utilized all the services (adequate ANC visit, delivered at health institution and post natal check up) for their last child. This study found that many socio-demographic variables such as age of women, number of children born, level of education, ethnicity, place of residence and wealth index are predicators of utilizing the maternal health services of recent child. Notably, higher level autonomy was associated with higher use of maternal health services [adjusted odds ratio (aOR) =1.40; CI 1.18–1.65].

**Conclusions:**

Utilization of maternal health services for the recent child among women is very low. The study results suggest that policy actions that increase women’s autonomy at home could be effective in helping assure good maternal health.

## Background

Maternal and Child mortality continues to be a major health problem in developing countries. One estimate made in 2015 puts the maternal death at 303000. Developing regions account for approximately 99 % of the global maternal deaths, with Sub-Saharan Africa alone accounting for roughly 66 %, followed by South Asia 22 % [1]. Improving maternal health is one of the eight Millennium Development Goals [2]. Despite various international efforts initiated to improve maternal health, about 287,000 women worldwide die each year as a result of complication arising from pregnancy and child birth [3]. A substantial body of research has examined the role of women’s autonomy on health and behavioral outcomes such as fertility [4], infant mortality [5] and child rearing and pregnancy care [6–8].

Reducing levels of maternal mortality and morbidity depends on increasing use of reproductive and maternal health services. Nepal has now met the MDG 5 target of reducing the maternal mortality ratio by ¾ by 2015 [9]. Maternal mortality ratio in Nepal has decreased significantly (901 per 1,00,000 live births in 1990 to 258 per 1,00,000 live births in 2015) over a time period, however, it is very high compared to other countries [1]. Although the government of Nepal has been implementing free delivery policy since 2009, it is one of the countries in Southern Asia where MMR is still high (258 per 100,000 live births) [1, 10]. Little and inequitable utilization of maternal health services is one of the reasons for such high number of maternal deaths in Nepal [11]. Fertility declined from 4.6 births per woman in the 1996 NFHS to 2.6 births per woman in the 2011 NDHS; a drop of two births per woman in the past 15 years. Nepal has also made an impressive increase in the use of modern contraceptive methods from 26 % in 1996 to 43 % in 2011 [10].

Women’s autonomy can be defined as the capacity and freedom to act independently. It encompasses women’s ability to formulate strategic choices, control resources, and participate in decision making. Researchers also identified some of the direct measures of women’s autonomy include access to and control over resources, participation in economic decisions, self-esteem, and mobility [5, 12, 13].

Regular antenatal care is very helpful in identifying and preventing adverse pregnancy outcomes. WHO has recommended that a woman should have at least four ANC visit. ANC is a key maternal service in improving a wide range of health outcomes for women and children. It is an opportunity to provide interventions for improving maternal nutrition and to encourage skilled attendance at birth and use of facilities for emergency obstetric care [14]. A birth delivered in health facilities is important for reducing deaths arising from complications of pregnancy. The expectation is that if complications arise during delivery in a health facility, a skilled attendant can manage the complication or refer the mother early to the next level of care. The postpartum period is particularly important for women, as during this period they may develop serious, life-threatening complications after delivery. Evidence has shown that a large proportion of deaths occur during this period, with postpartum hemorrhage being an important cause [10].

Researchers have shown that higher status for women correlates positively with their health [15] and that of their children [5]. Study found that positive associations between women’s empowerment and lower fertility, longer birth interval and lower rates of unintended pregnancy [16]. Studies also found that use of maternal health care services is influenced by women’s roles in decision-making [17–19].

This paper examines whether women’s autonomy is associated with maternal health service utilization (adequate ANC visit, delivered at health facilities and postnatal check up) in the context of Nepal. It is found that women in Nepal are disadvantaged compared to men in terms of their access to assets, employment, health care, and education. Since the social status and level of autonomy of Nepalese women is low, their status at the household level needs to be further explored in terms of health services utilization, which has a direct impact on maternal and infant morbidity and mortality. This relationship clearly warrants further attention, particularly in settings such as Nepal, where maternal and child health utilization is low [10]. We hypothesize that women with higher autonomy are more likely to seek health care during pregnancy, delivery and Post natal care than those with lower autonomy. This paper aims to close the knowledge gap in the literature with regards to a society in which women suffer gross disadvantages in the context of a patriarchal culture, which in turn can help guide reproductive health program planners and policy makers to understand various factors influencing maternal health service utilization and to assist in implementation of reproductive health programs that will decrease maternal morbidity and mortality.

## Methods

### Data source

This paper uses data from the Nepal Demographic and Health Survey, 2011, a nationally representative sample survey. The 2011 NDHS was carried out under the aegis of the Population Division of the Ministry of Health and Population. The primary purpose of this survey was to provide current and reliable data on fertility and family planning, child mortality, children’s nutritional status, utilization of maternal and child health services, domestic violence, and knowledge of HIV/AIDS. The study protocol was approved by the Nepal Health Research Council and the ICF Macro Institutional Review Board in Calverton, Maryland, USA. All respondents had provided verbal informed consent to be interviewed prior to data collection. Therefore, an independent ethical approval was not required. For this study, we used publicly available dataset from the measure DHS website [20].

### Study participants

Interviews were completed for 12,674 women of reproductive age. However, this analysis is confined to women who had given at least one singleton birth in the five-year preceding the survey (*n* = 4,148). Detail methodology and questionnaire used in the survey can be found in the published report of Nepal demographic and health survey [10].

### Data analysis

Association between women’s autonomy and maternal health service utilization (adequate ANC care, delivery at health facility and postnatal care) was assessed via bivariate analysis using chi-square tests. Then logistic regression was used to assess the net effect of women’s autonomy on maternal health service utilization after controlling for several other independent variables. Two models were run in the analysis. The first model contained women’s autonomy only and dependent variable i.e. maternal health service utilization for ANC, delivery and PNC. In the second model, i.e. full model added the other socio-demographic and economic characteristics such as age of women, total children, ethnicity, education, religion, place of residence, wealth index and women’s involvement in community level activities.

### Dependent variable

Dependent variable in this paper is made from composite index of the variables related to utilization of maternal health services (4 or more ANC visit, delivery at health facility, and postnatal care) of recent child within the past five years preceding the survey. The value of 1 is assigned if the respondents had visited 4 or more ANC, delivered at health facilities and had visited for post natal check up; and 0 was assigned if respondents did not utilize all these services for the recent child.

### Independent variables

In the present study, key explanatory variable was made from the indicators related to women’s autonomy from the standard DHS questionnaire. Women’s autonomy refers to women’s decision-making autonomy, which was measured based on responses to “Who makes the following decisions in (respondent’s) household about: 1) obtaining health care for yourself; 2) large household purchases; and 3) visits to family or relatives?” Response options were: a) respondent alone; b) respondent and husband/partner; c) respondent and other person; d) husband/partner alone; e) someone else; f) other. The value of 1 is assigned if the response was (a), (b), or (c), that is, involvement of the respondent, or else 0, for no involvement of the respondent. The other independent variables included in this study were demographic and socioeconomic variables such as age, number of children born, caste/ethnicity, religion, place of residence, wealth status of households, involvement in community group. In order to avoid bias towards the over-sampled sub populations, sample weight were applied for all estimates (mean and percentages). After assessing multicollinearity in the variables, it was found that the variables ‘age of women’ and ‘number of children ever born’ were highly correlated. So the variable ‘number of children ever born’ was not entered in the logistic regression model.

## Results

Two-fifth of the married women who had given birth in the five years preceding the survey (40 %) was youth aged 15–24. More than a fifth of them had four or more children. A considerable proportion of them (37 %) were from Janajati ethnic group followed by Brahmin/Chhetri (31 %). Only about two in five women (36 %) had secondary or above education. An overwhelming majority of women believe Hindu religion (83 %), and live in rural area (90 %). Involvement in community group among women was low (39 %). Similarly, women’s autonomy regarding household decision was low in the country. Only less than two in five women (38 %) had autonomy on household decisions (Table 1).

**Table 1:** Background characteristics of respondents

	Percent	Number
Age group		
Less than 25 years	40.1	1662
25–29 years	31.6	1310
30 years or above	28.4	1176
Total children		
One	31.4	1302
Two	28.0	1161
Three	17.7	733
Four or more	22.9	952
Ethnicity		
Brahmin/Chhetri	30.9	1282
Janajati	36.7	1523
Dalit	16.5	683
Other	15.9	660
Education		
No education	43.9	1822
Primary	20.1	835
Secondary or above	36.0	1491
Religion		
Hindu	83.0	3444
Non-Hindu	17.0	704
Place of residence		
Urban	10.1	418
Rural	89.9	3730
Wealth index		
Poor	45.3	1878
Middle	21.0	873
Rich	33.7	1397
Involvement in community group		
Not involved in any community group	60.9	2526
Involvement in community group	39.1	1622
Women’s autonomy		
Not involved in all three household decisions	62.1	2575
Has any say in all three household decisions	37.9	1573
Total	100.0	4148

A half of the women who had given birth in the past five years preceding the survey had 4 or more ANC check up for their last birth. Similarly, 40 % of the women had delivered their last child at the health facilities. Slightly higher than two in five women (43 %) reported that they had post natal check up for their last birth. Notably, only a fourth women (27 %) had utilized the all above services (4 or more ANC visit, delivered last birth at health facilities and postnatal check up) for the most recent live birth in the 5 years preceding the survey (Table 2).

**Table 2:** Maternal health service utilization for the most recent live birth in the five year preceding the survey

	Percent	Number
Number of ANC visits		
less than 4 visit	49.9	2071
4 or more visits	50.1	2077
Place of delivery		
Home	59.8	2480
Health facilities	40.2	1668
Postnatal check up		
No	56.8	2357
Yes	43.2	1791
Utilization of all above services		
No	72.8	3021
Yes	27.2	1127
Total	100.0	4148

Women’s autonomy as measured by household decision making was associated with maternal health service utilization. Maternal health service utilization was higher among those women who had autonomy in household decisions (32 %) compared to those who did not have autonomy (24.5 %). A significantly higher percentage of women who were less than 30 years (30 %) had utilized maternal health services compared with those who had 30 years and above (20 %). About half women who had one child while only less than tenth women who had four or more children (9 %) utilized maternal health services. A higher percentage of women from Brahmin/Chhetri (39 %) compared with Janajati (25 %) and Dalit (17 %) had utilized maternal health services. Similarly, a higher percentage of women who had secondary or above education (51 %), who live in urban area (57 %), who believed Hindu religion (29 %), who were from rich household (53 %) and who involved in community group utilized maternal health services compared with other categories (Table 3).

**Table 3:** Background characteristics of respondents by maternal health service utilization *(Adequate ANC check up, delivered in health facility and PNC check up)* for the most recent live birth in the five year preceding the survey

	Utilized all maternal health services	Not utilized all maternal health services	N
Women’s autonomy ***			
Not involved in all three household decisions	24.5	75.5	2575
Has any say in all three household decisions	31.6	68.4	1573
Age group***			
Less than 25 years	30.0	70.0	1662
25–29 years	30.1	69.9	1310
30 years or above	19.9	80.1	1176
Total children ***			
One	43.7	56.3	1302
Two	29.5	70.5	1161
Three	17.1	82.9	733
Four or more	9.4	90.6	952
Ethnicity ***			
Brahmin/Chhetri	38.7	61.3	1282
Janajati	24.6	75.4	1523
Dalit	17.3	82.7	683
Other	20.8	79.2	660
Education ***			
No education	10.4	89.6	1822
Primary	21.5	78.5	835
Secondary or above	50.8	49.2	1491
Religion ***			
Hindu	28.8	71.2	3444
Non-Hindu	19.2	80.8	704
Place of residence ***			
Urban	57.1	42.9	418
Rural	23.8	76.2	3730
Wealth index ***			
Poor	10.3	89.7	1878
Middle	22.4	77.6	873
Rich	52.7	47.3	1397
Involvement in community group **			
Not involved in any community group	25.7	74.3	2526
Involvement in community group	29.5	70.5	1622
Total	27.2	72.8	4148

*** Significant at *p* < 0.001; ** = *p* < 0.01 and * = *p* < 0.05

In the first model, women’s autonomy has positive and statistically significant effect on maternal health service utilization.. The results indicate that women who have involved in household decisions were more likely utilized all maternal health service (OR = 1.42) compared with those who did not have autonomy. Women’s autonomy variable retained its significance even after inclusion of demographic and socio-economic variables in the model 2. The slighly reduction on the odds ratio of women’s autonomy (adjusted odds ratio = 1.40) after inclusion of demographic and social economic variables were also important predictors of utilization of maternal health service. Model 2 further explained that women aged 30 years or above were less likely to use maternal health services (aOR = 0.77) compared to youth women aged 15–24 keeping all other variables constant in the model. Similarly, women from Janajati were less likely to utilize the services (aOR = 0.75) than women from Brahmin/Chhetri. Likewise, women who lived in rural women were less likely to use maternal health services (aOR = 0.51) than women from Urban. On the other hand, women who have primary and secondary or above education were about 2 times (aOR = 1.8) and 4 times (aOR = 4.06) respectively more likely to use maternal health service than illiterate women. Similarly, women who were from middle and rich households were about 1.9 and 4.5 times respectively more likely to use maternal health services than women from poor households (Table 4).

**Table 4:** Adjusted odds ratios (aOR) from multivariable logistic regression assessing the likelihood of utilizing maternal health services of recent child within the past five years preceding the survey by women’s autonomy and selected social and demographic characteristics

Selected predicators	Model I			Model II		
	OR	95 % CI		OR	95 % CI	
Women’s autonomy						
Not involved in all three household decisions (ref.)	1.00			1.00		
Has any say in all three household decisions	1.42***	1.24 -	1.64	1.40***	1.18 -	1.65
Demographic and socio-economic variables						
Age group						
Less than 25 years (ref.)				1.00		
25–29 years				0.95	0.78-	1.15
30 years or above				0.77*	0.62-	0.96
Ethnicity						
Brahmin/Chhetri (ref.)				1.00		
Janajati				0.75**	0.61-	.91
Dalit				0.87	0.66-	1.14
Other				0.78	0.59-	1.03
Education						
No education (ref.)				1.00		
Primary				1.81***	1.42-	2.31
Secondary or above				4.06***	3.25-	5.07
Religion						
Hindu (ref.)				1.00		
Non-Hindu				0.81	0.64-	1.04
Place of residence						
Urban (ref.)				1.00		
Rural				0.51***	0.40-	0.65
Wealth index						
Poor (ref.)				1.00		
Middle				1.93***	1.53-	2.42
Rich				4.47***	3.61-	5.53
Involvement in community group						
Not involved in any community group (ref.)				1.00		
Involvement in community group				1.02	0.86-	1.20
*Constant*	0.324***			0.165***		
*- 2 log likelihood*	*4828.3*			*3799.5*		
*Cox & Snell R Square*	*0.006*			*0.224*		

*** Significant at *p* < 0.001; ** = *p* < 0.01 and * = *p* < 0.05

## Discussion

This study assessed the effect of women’s autonomy on the maternal health service utilization among women in Nepal. Three measures of maternal health service utilization were considered: 4 or more ANC visit, delivery at health facility, and postnatal care. Women’s autonomy is measured by women’s decision-making autonomy on obtaining health care for her; large household purchases and visits to family or relatives. Results show that maternal health service utilization is very low in Nepal, thus indicating an unmet need to be addressed by maternal and child health programs.

As do many other studies, this study also shows that educated women were more likely to utilize maternal health care services than illiterate women. It could be that educated women are more capable of accessing available health facilities and that they are able to greatly change the traditional balances of power and autonomy in familial relationships, with profound effects on health care. Another reason could be that schools are institutions that transform young girls into empowered, assertive, and confident women [21].

This study showed that maternal health service utilization is significantly higher among those women who had autonomy in decisions regarding their household activities compared to those who were not. This study is similar to the study that suggests use of maternal health care services is influenced by women’s roles in decision-making [19]. A possible explanation could be that women who have autonomy in decision making are more likely to have a higher level of autonomy on health care, which might lessen their reproductive behavior risks [22]. Another study has confirmed that a women’s control over household resources (ability to keep money aside) has a significant positive effect on both the demand for prenatal care and the probability of hospital delivery [23].

There are some limitations in the interpretation of the results of this study. Women’s autonomy is a complex phenomenon that cannot be completely measured by a few household decision-making indicators. Similarly, as pointed out previously, we restricted our subjects to women who had given at least one singleton birth in the five-year preceding the survey, so our results regarding the utilization of maternal health services should be generalized with care. Because the cross-sectional design of the study and all of the items analyzed in the logistic regression analysis came from information at the time of survey, the analysis can only provide evidence of statistical association between those items and utilization of health services and cannot show cause-effect relationships. Comparisons between surveys should also be interpreted with caution because quality of data and sample coverage varies.

## Conclusions

The findings of this study highlight the need for initiatives to improve women’s position in Nepal, both to attain gender equality and to promote women’s reproductive health. Women’s autonomy is a strong predictor among many other predictors of maternal health service utilization. Mothers’ decision-making power appears to be the most powerful predictors among many others for increasing maternal health service utilization. The study results suggest that policy actions that increase women’s autonomy at home could be effective in helping assure good maternal health.

### Ethics approval and consent to participate

The study protocol was approved by the Nepal Health Research Council and the ICF Macro Institutional Review Board in Calverton, Maryland, USA. All respondents had provided verbal informed consent to be interviewed prior to data collection.

### Consent for publication

Not applicable

### Availability of data and materials

The data used are publicly available from the Measure DHS site.
